# A New Candidate Supporting Drug, Rikkunshito, for the QOL in Advanced Esophageal Cancer Patients with Chemotherapy Using Docetaxel/5-FU/CDDP

**DOI:** 10.1155/2011/715623

**Published:** 2011-08-17

**Authors:** Junichi Seike, Toru Sawada, Naoya Kawakita, Yota Yamamoto, Yasuhiro Yuasa, Hiromichi Yamai, Hirokazu Takachi, Takahiro Yoshida, Akira Tangoku

**Affiliations:** Department of Thoracic, Endocrine Surgery and Oncology, Institute of Health Bioscience, The University of Tokushima Graduate School, Tokushima 770-8503, Japan

## Abstract

*Purpose*. Docetaxel/5-FU/CDDP (DFP) therapy is a useful treatment for advanced esophageal cancer. However, adverse reactions such as chemotherapy-induced nausea and vomiting (CINV) interfere often with continuation of the chemotherapy. We investigated the efficacy of rikkunshito (TJ-43) on CINV. *Methods*. Nineteen patients who were going to undergo DFP therapy were enrolled. They were assigned to the following two groups: a TJ-43-treated group and -nontreated group. The following parameters were compared between the 2 groups: (1) the frequency of symptoms occurred, (2) vomiting, nausea, and anorexia score, and (3) QOL score. *Results*. The incidence of symptoms was lower in the TJ-43-treated group than that in the control group. The nausea score of the TJ-43-treated group was significantly lower than that of the control group. In the QOL score, the mood score and the ADL score decreased significantly in the control group. *Conclusion*. We recommend TJ-43 administration in patients undergoing DFP chemotherapy.

## 1. Introduction

Esophageal cancer is a highly malignant neoplasm with poor prognosis. The mortality of this disease is 9.0 per 100,000 (15.7 per 100,000 in males, 2.6 in females), representing 3.4% (4.9% in males, 1.3% in females) of all deaths by malignant neoplasm in 2006. Mortality has increased slightly over the past two decades despite recent improvements in diagnosis and treatment [1]. The esophagus has complicated relations not only with the nearby organs such as trachea, lung, and aorta but also with the lymphatic tissue network. Therefore, the cancer generated at the esophagus shows easily lymph node metastasis in the wide range from the cervical region to the abdominal region even when it is a superficial cancer [2]. This is why surgical intervention accompanied by extensive lymph node dissection has been performed for the radical treatment of esophagus cancer [3]. Since the invasion accompanying such radical surgery gives marked influences on the postoperative quality of life (QOL) [4], the importance of multidisciplinary approach including surgery/chemotherapy/radiotherapy is being recognized more and more to achieve successful radical therapy. Japan clinical oncology group (JCOG) conducted a phase II study of chemoradiotherapy in patients with stage II, III esophageal carcinoma (ESCC): (JCOG9906 study), but the result showed lesser than the surgical results of Japanese high-volume centers [5]. The effect of chemotherapy on esophagus cancer has already been demonstrated [6], and it was recently reported that preoperative chemotherapy is more effective than postoperative chemotherapy [7]. We have actively performed DFP therapy as preoperative chemotherapy and obtained favorable results, but leukocytopenia and alopecia and also gastrointestinal adverse reactions such as vomiting, nausea, and anorexia (so-called chemotherapy-induced nausea and vomiting: CINV) interfere often with continuation of the chemotherapy [8]. To alleviate for CINV, a steroid preparation, a 5-HT3 antagonist, and a gastrointestinal complaint-treating agent are used, but these drugs are not satisfactory in the light of efficacy and safety, and there is also a problem in the aspect of medical economy. 

 Recently in Japan, the kampo preparation rikkunshito (TJ-43) having a gastrointestinal motility-improving effect is often used for the treatment of vomiting, nausea, or anorexia. Tomono et al. reported the effect of TJ-43 on retching occurring during breast cancer chemotherapy [9]. It is therefore expected that TJ-43 would exert an alleviating effect for the CINV caused by DFP therapy. However, few papers have clearly referred to the effect of TJ-43 on vomiting, nausea, or anorexia occurring during chemotherapy. In this study, for the purpose of improving the QOL of patients undergoing chemotherapy, we investigated the efficacy and safety of TJ-43 in the treatment of adverse reactions (vomiting, nausea or anorexia) to DFP therapy in patients with advanced esophagus cancer.

## 2. Methods

This study was approved by the Ethics Committee of Tokushima University Hospital and conducted in compliance with the ethical principle of the Helsinki Declaration.

### 2.1. Study Patients

In the period from August 2008 to March 2009, 19 patients with advanced esophagus cancer (age: 52–79 years, disease stage: II or III) who were going to undergo DFP therapy submitted written informed consent to participate in this study after receiving sufficient explanations about the contents of this study. The ECOG Performance Scale needed to be 0–2 in these patients. The exclusion criteria were as follows: (1) patients who had undergone gastrectomy or endoscopic gastrostomy, (2) patients with renal dysfunction, cardiac dysfunction, or bone-marrow dysfunction, (3) patients with serious complication such as cardiac failure or acute inflammatory disease, (4) female patients who were pregnant, planning pregnancy, or breastfeeding, and (5) patients taking other kampo drug excluding rikkunshito.

### 2.2. Drugs

Rikkunshito, one of traditional Japanese medicines, which has been approved for medicinal use by the Japanese Ministry of Health and Welfare, is extract granules for Ethical Use (Tsumura and Co., Product number TJ-43, 7.5 g), containing 4.0 g of dried extract obtained from mixed crude drugs in the following ratio: JP Atractylodes Lancea Rhizome, 4.0 g; JP Ginseng, 4.0 g; JP Pinellia Tuber, 4.0 g; JP Poria Sclerotium, 4.0 g; JP Jujube, 2.0 g; JP Citrus Unshiu Peel, 2.0 g; JP Glycyrrhiza, 1.0 g; and JP Ginger, 0.5 g. Subjects took 2.5 g of rikkunshito three times a day before each meal.

### 2.3. Study Design

The basic chemotherapy in this study was DFP therapy, which was performed according to the regimen specified at this hospital for advanced esophagus cancer. In detail, CDDP 10 mg/body was intravenously infused on days 1–5, 5-FU 370 mg/m^2^ was intravenously infused on days 1–5, docetaxel 25 mg/m^2^ was intravenously infused on day 1 and day 8 in each cycle, and this cycle was repeated 4 times. This study was conducted in the period from day 1 to day 14. As antiemetic drugs, azasetron 10 mg/day was intravenously infused on days 1–5 and dexamethasone 8 mg was intravenously infused on Day 1.

Subjects were randomly assigned to the TJ-43-treated group and the TJ-43-non-treated group (the control group). In the TJ-43-treated group, TJ-43 was orally administered from Day 1 for 2 weeks. All the subjects were inquired about the symptoms on Days 1–5, Day 8, and Day 14. The investigators who assessed severity and QOL were not informed which group the patients belonged to.

### 2.4. Efficacy Evaluation

The primary index was the change in each symptom (vomiting, nausea, or anorexia) 2 weeks after the TJ-43 treatment. The symptom severity was evaluated with CTCAE Version 3.0 on days 1–5, day 8, and day 14. For comparison between the 2 groups, the CTCAE grade was scored according to the following rules: no symptoms: 0 point, and CTCAE grade 1–4: 1 point-4 point.

**Figure 1:**
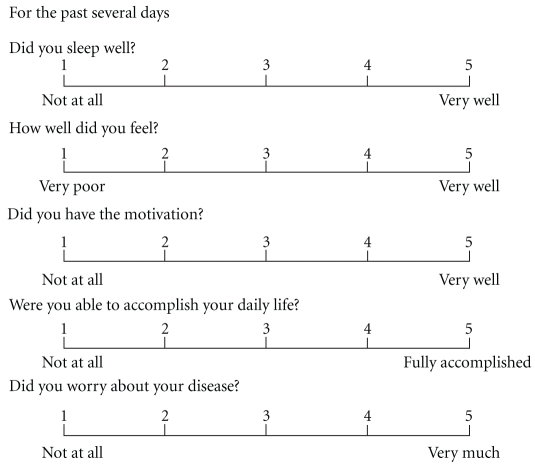
Interview sheet of QOL.

As the secondary index, the effect of TJ-43 treatment on QOL was evaluated on Day 1 and Day 14. Five items of sleep, mood, volition, activity of daily living (ADL), and anxiety feeling were set for QOL scoring. Each item was scored on the five-grade scale of 1–5 according to QOL-ACD [10] (Figure 1). Almost patients filled out question paper by themselves. But some patients could not fill it because of bad condition; so the investigator asked symptoms showing the interview sheet and filled it for them. The assessment was done by the doctor who did not participate in this study.

### 2.5. Safety Evaluation

An adverse event was defined as any unfavorable or unintended sign, whether or not considered to be causally related to the study drug, and was recorded in the medical record. On day 14, the patients answered the standardized question: “Have you had any health problems since you started to take the study drug”?

### 2.6. Statistical Analysis

In order to summarize the subject information before the treatment, the subject background factors and the summary statistics of the evaluation items on Day 1 were obtained for all the subjects assigned. One subject who deviated from the age-related inclusion criterion was not included in the efficacy analysis. The differences between Day 1 and Day 14 were subjected to calculation of summary statistics and intergroup comparison by Wilcoxon's rank sum test. The differences were tested for significance with a two-sided significance level of 5%. The intragroup comparison between Day 1 and Day 14 was performed by Wilcoxon's signed rank test. No adjustment was made for multiple tests.

## 3. Results

**Figure 2:**
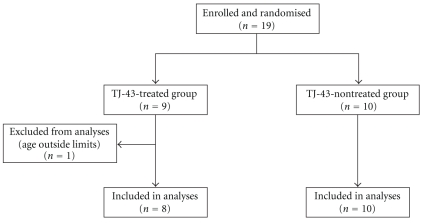
Enrollment and randomization of study patients.

No subjects reported vomiting, nausea, or anorexia before the assignment. One subject of the TJ-43 treated group was excluded from evaluation due to deviation from the age-related inclusion criterion (Figure 2). 

There were no differences between the two groups in male/female ratio, age, and BMI of the subjects (Table 1).

### 3.1. Incidence of Symptoms

Table 2 shows the number and rate of subjects who reported vomiting, nausea, or anorexia in each group. The incidence of vomiting, nausea, or anorexia was lower in the TJ-43 treated group than that in the TJ-43 non-treated group. In the TJ-43-treated group, 3 of 8 subjects presented with vomiting, nausea or anorexia on Day 14. On the other hand, in the TJ-43 non-treated group, 8 of 10 subjects had vomiting, nausea, or anorexia on Day 14, but the symptom incidence was not significantly different between the 2 groups.

### 3.2. Time-Course Changes in Score of Vomiting, Nausea, or Anorexia

**Figure 3:**
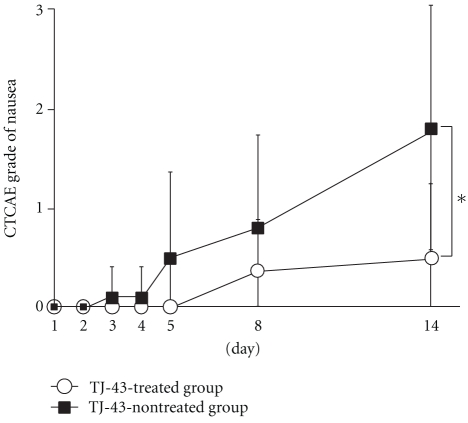
Time-course changes in score of nausea. Each plot refers to mean ± SD. ∗: Significant difference of the score deterioration between the TJ-43 treated group and the TJ-43 non-treated group (*P*  < 0.05, Wilcoxon's rank sum test).

**Figure 4:**
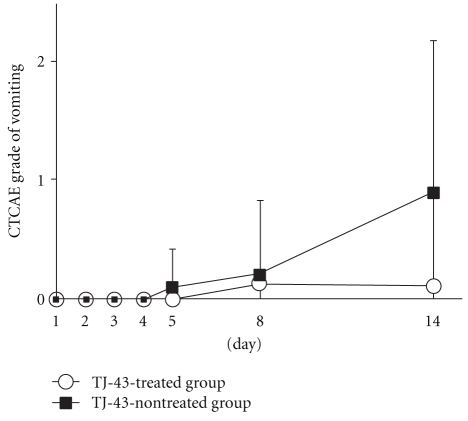
Time-course changes in score of vomiting. Each plot refers to mean ± SD. There were no significant differences in score change between the TJ-43 treated group and the TJ-43 non-treated group (Wilcoxon's rank sum test).

**Figure 5:**
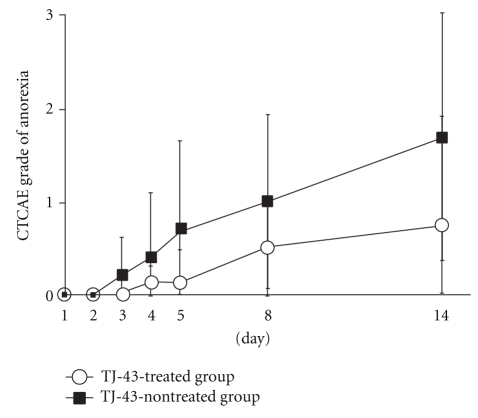
Time-course changes in score of anorexia. Each plot refers to mean ± SD. There were no significant differences in score change between the TJ-43 treated group and the TJ-43 non-treated group (Wilcoxon's rank sum test).

The increase of nausea score was significantly suppressed in the TJ-43 treated group as compared with TJ-43 non-treated group (0.50 ± 0.76 versus 1.80 ± 1.23 on day 14) (Figure 3). The increases of vomiting score and anorexia score tended to be suppressed in the TJ-43 treated group as compared with the TJ-43 non-treated group, but no significant inter-group difference was observed (Figures 4 and 5).

### 3.3. Time-Course Changes in Score of QOL

**Figure 6:**
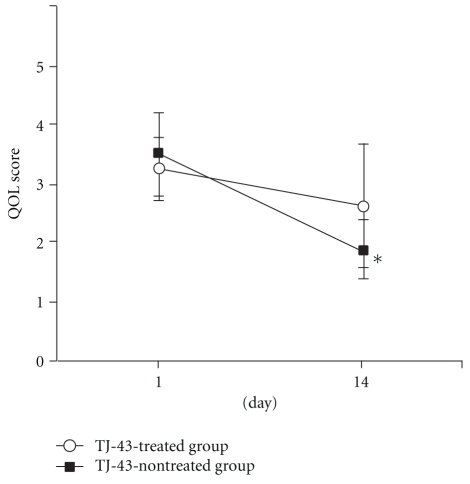
Change in score of mood. Each plot refers to mean ± SD. *Significant difference between the score on day 1 and day 14 in the TJ-43 non-treated group (*P* < 0.05, Wilcoxon's signed rank test).

**Figure 7:**
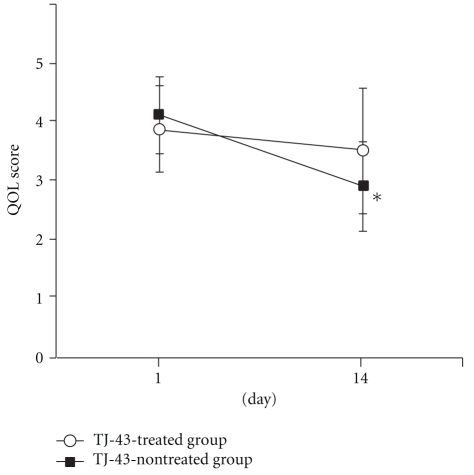
Changes in score of ADL. Each plot refers to mean ± SD. *Significant difference between the score on day 1 and day 14 in the TJ-43 non-treated group (*P*  < 0.05, Wilcoxon's signed rank test).

**Figure 8:**
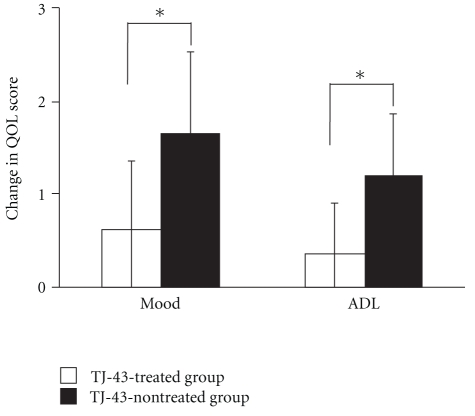
Change in QOL score of mood or ADL after the 2-week treatment. Each column represents the mean ± SD. *Significant difference of the change in QOL score between the TJ-43 treated group and the TJ-43 non-treated group (*P* < 0.05, Wilcoxon's rank sum test).

Among the QOL scores, the mood score decreased significantly from 3.50 ± 0.53 on day 1 to 1.89 ± 1.05 on day 14 in the TJ-43 non-treated group but showed no change in the TJ-43 treated group (Figure 6). Similarly, the ADL score decreased significantly from 4.10 ± 0.74 on Day 1 to 2.89 ± 1.05 on Day 14 in the TJ-43 non-treated group (Figure 7). The differences between Day 1 and Day 14 in mood score and ADL score were significantly lower in the TJ-43 treated group than those in the TJ-43 non-treated group (Figure 8). The changes in scores of sleep, volition, and anxiety feeling were not different between the TJ-43 treated group and the TJ-43 non-treated group. 

### 3.4. Adverse Events

In the study period, none of the subjects developed any potential adverse reactions to TJ-43.

### 3.5. Therapeutic Effect of DFP Therapy

Looking at the therapeutic effect of DFP therapy (Cycle 1), CR was seen in 2 subjects, PR in 5 subjects, and SD in 1 subject in the TJ-43 treated group, and CR in 3 subjects, PR in 5 subjects, and SD in 2 subjects in the TJ-43 non-treated group.

## 4. Discussion

This is the first prospective randomized study that demonstrated the efficacy of TJ-43 against CINV accompanying DFP therapy in patients with advanced esophagus cancer.

Esophagus cancer is one of the malignant tumors encountered frequently and ranked seventh among the cancer-related causes of death in the world [11]. It is one of the intractable cancers. Esophagectomy accompanied by extensive lymph node dissection is the standard therapy, but the 5-year survival rate achieved by this surgery alone was reported to be only 10%–20% in the advanced cases [12–14]. In the advanced cases, the possibility of recurrence is high even after radical resection, and the therapeutic effect of surgical therapy alone is insufficient. It is therefore necessary to perform effective perioperative management therapy for achieving successful radical therapy, and as the standard therapy against esophagus cancer, FP therapy using CDDP and 5-FU was proposed [15, 16]. Furthermore in recent years, FP therapy added with docetaxel (DFP therapy) was reported to give a higher response rate [17–19] and we also obtained favorable results in patients with esophagus cancer [20, 21]. However, whereas DFP therapy is more effective than FP therapy, DFP therapy often induces not only leukocytopenia and alopecia but also gastrointestinal symptoms such as vomiting, nausea, and/or anorexia. CINV are common adverse reactions of chemotherapy, but they are considered by patients to be one of the most distressing concerns of cancer treatment [22, 23]. The failure in prevention of CINV in the first cycle of chemotherapy increases the patient's resistance to the chemotherapy leading to abandonment of cancer therapy [24, 25]. Therefore, CINV control is the key point for successful completion of chemotherapy and QOL maintenance in the patients. CINV is roughly classified into acute, delayed, and anticipatory types depending on the onset timing of vomiting [26]. The symptoms occurring in the first 24 hours after starting the chemotherapy are defined as acute CINV and can be controlled with an antiemetic drug. On the other hand, the delayed CINV occurring more than 24 hours after starting the chemotherapy show a lower incidence as compared with the acute CINV but are difficult to control [27]. In order to control the delayed CINV, a dopamine antagonist, a steroid, a 5-HT3 antagonist and an NK1 receptor antagonist are used alone or in combination in a preventive manner from before starting the chemotherapy [28], but the effect is not necessarily satisfactory. Then, the second-generation 5-HT3 antagonist and NK1 receptor antagonist have been used only for a short period yet in Japan, and besides, these drugs are expensive, with problem in view of medical economics. To complete chemotherapy while maintaining the patient's QOL, it is desirable to establish a convenient and effective treatment method.

This time, we focused on a kampo drug TJ-43, which is frequently used not only against functional dyspepsia, gastroesophageal reflux disease, and gastrointestinal symptoms after surgery but also against gastrointestinal symptoms such as nausea and vomiting induced by a psychotropic drugs or anticancer drugs [29–33]. So, we investigated the possibility of TJ-43 as an antiemetic drug. As a result, the incidences of CINV or anorexia tended to be lower in the TJ-43 treated group, and especially TJ-43 treatment significantly suppressed the incidence of nausea. Furthermore, the QOL deterioration in terms of mood or ADL was suppressed by the TJ-43 treatment. These results suggested that oral administration of TJ-43 would alleviate the unpleasant symptoms in patients undergoing chemotherapy. The fact that the difference in intensity of CINV between the TJ-43 treated group and the TJ-43 non-treated group became greater with increasing time after starting the treatment suggested the efficacy of TJ-43 against the delayed CINV refractory to a 5-HT3 antagonist.

As the action mechanism supporting the clinical effect of TJ-43, Tominaga et al. clarified that TJ-43 enhances gastric motility through the 5-HT3 receptor-antagonistic effect and speculated that the effect is mediated by a signal transmission route different from that for 5-HT3 receptor-antagonist [34]. If TJ-43 has such specific effect, it may be possible to explain the reason why TJ-43 can alleviate the CINV induced by serotonin in the case of cisplatin, and so forth; especially the delayed CINV are resistant to a 5-HT3 antagonist. To investigate usefulness of TJ-43 is required for different regimens of chemotherapy and for variety of cancer types hereafter.

Our study was performed as a pilot study to conduct a future large-scale randomized controlled trial. There may be a limitation of statistical confidence in this study, because number of subjects was small. However, even among the small number of subjects, there was a significant difference. Therefore, we consider that the results are worth reporting. In this study, the efficacy of TJ-43 against CINV was shown in esophagus cancer patients undergoing preoperative chemotherapy (DFP therapy). The add-on treatment with TJ-43 not only suppressed the increase of nausea score but also maintained the patient's mood and ADL. We propose to use TJ-43 together with a 5HT3 antagonist, a steroid, an NK1 receptor antagonist, and so forth as one of the anti-CINV treatment strategies in patients undergoing chemotherapy.

## Figures and Tables

**Table 1:** Demographic and medical characteristics of the study patients.

	TJ-43-treated group (*n* = 9)	TJ-43-nontreated group (*n* = 10)
Sex (male/female)	8/1	7/3
Age (mean ± SD)	63 ± 6.2	68.1 ± 7.3
BMI (mean ± SD)	21.9 ± 1.9	21.2 ± 3.3
Clinical stage		
II A	1	1
II B	1	0
III	2	7
IV	5	2
Performance status		
0	9	9
1	0	1

**Table 2:** Frequency of vomiting, nausea, and anorexia at 14 days.

		TJ-43 treated group *N* (%)	TJ-43 nontreated group *N* (%)
Vomiting	Not reported	7 (87.5)	6 (60.0)
	Reported	1 (12.5)	4 (40.0)
Nausea	Not reported	5 (62.5)	2 (20.0)
	Reported	3 (37.5)	8 (80.0)
Anorexia	Not reported	5 (37.5)	3 (30.0)
	Reported	3 (62.5)	7 (70.0)

## Abbreviations

TJ-43: Rikkunshito
DFP: Docetaxel/5-FU/CDDP
QOL: Quality of life
JCOG: Japan clinical oncology group
CINV: Chemotherapy-induced nausea and vomiting
ADL: Activities of daily living.
